# Differential Recognition of Clinically Relevant *Sporothrix* Species by Human Mononuclear Cells

**DOI:** 10.3390/jof9040448

**Published:** 2023-04-06

**Authors:** Laura C. García-Carnero, Iván Martínez-Duncker, Manuela Gómez-Gaviria, Héctor M. Mora-Montes

**Affiliations:** 1 Departamento de Biología, División de Ciencias Naturales y Exactas, Campus Guanajuato, Universidad de Guanajuato, Noria Alta s/n, col. Noria Alta, C.P., Guanajuato 36050, Gto., Mexico; laura_cgc@hotmail.com (L.C.G.-C.); manuela.gomezg8@gmail.com (M.G.-G.); 2 Laboratorio de Glicobiología Humana y Diagnóstico Molecular, Centro de Investigación en Dinámica Celular, Instituto de Investigación en Ciencias Básicas y Aplicadas, Universidad Autónoma del Estado de Morelos, Cuernavaca 62209, Mor., Mexico; duncker@uaem.mx

**Keywords:** fungal cell wall, protein glycosylation, *N*-linked glycans, *O*-linked glycans, innate immune sensing

## Abstract

Sporotrichosis is a human and animal fungal infection distributed worldwide that is caused by the thermodimorphic species of the *Sporothrix* pathogenic clade, which includes *Sporothrix brasiliensis*, *Sporothrix schenckii*, and *Sporothrix globosa*. The cell wall composition and the immune response against the *Sporothrix* species have been studied mainly in *S. brasiliensis* and *S. schenckii*, whilst little is known about the *S. globosa* cell wall and the immune response that its components trigger. Therefore, in this study, we aimed to analyze the cell wall composition of *S. globosa* in three morphologies (germlings, conidia, and yeast-like cells) and the differences in cytokine production when human peripheral blood mononuclear cells (PBMCs) interact with these morphotypes, using *S. schenckii* and *S. brasiliensis* as a comparison. We found that *S. globosa* conidia and yeast-like cells have a higher cell wall chitin content, while all three morphologies have a higher β-1,3-glucan content, which was found most exposed at the cell surface when compared to *S. schenckii* and *S. brasiliensis*. In addition, *S. globosa* has lower levels of mannose- and rhamnose-based glycoconjugates, as well as of *N*- and *O*-linked glycans, indicating that this fungal cell wall has species-specific proportions and organization of its components. When interacting with PBMCs, *S. brasiliensis* and *S. globosa* showed a similar cytokine stimulation profile, but with a higher stimulation of IL-10 by *S. globosa*. Additionally, when the inner cell wall components of *S. globosa* were exposed at the surface or *N*- and *O*-glycans were removed, the cytokine production profile of this species in its three morphotypes did not significantly change, contrasting with the *S. schenckii* and *S. brasiliensis* species that showed different cytokine profiles depending on the treatment applied to the walls. In addition, it was found that the anti-inflammatory response stimulated by *S. globosa* was dependent on the activation of dectin-1, mannose receptor, and TLR2, but not TLR4. All of these results indicate that the cell wall composition and structure of the three *Sporothrix* species in the three morphologies are different, affecting their interaction with human PBMCs and generating species-specific cytokine profiles.

## 1. Introduction

Fungal infections are a public health problem of particular concern due to the associated high mortality rate that exceeds 50% [1]. Sporotrichosis is a subcutaneous mycosis with worldwide distribution that is prevalent in tropical and subtropical regions, predominating in North and South America, Asia, Australia, and some African countries [2,3,4,5]. The disease, which affects humans and other mammals, has different clinical manifestations, and most frequently, the lesions are restricted to the skin, subcutaneous tissue, and lymphatic vessels [6,7]. However, in immunocompromised patients, sporotrichosis can be fatal since the fungus can generate a deep-seated infection that spreads to other organs [6,8].

The etiological agents of the infection belong to the *Sporothrix* pathogenic clade, which includes the species *Sporothrix schenckii*, *Sporothrix brasiliensis*, *Sporothrix globosa*, and *Sporothrix luriei*; these first three species are the ones more frequently isolated from both clinical and veterinary cases [7,9,10]. The species that belong to this clade are thermodimorphic fungi, demonstrating a saprophytic phase that grows at 25–28 °C as conidia and germling-producer mycelia, and the parasitic phase, which grows at 34–37 °C as yeast-like cells [4,5]. However, the filament form has also been observed in human and animal samples from sporotrichosis and in biopsies from experimental infections [11,12,13]. Therefore, this morphology is as relevant as the yeast-like cells during the host invasion. The *Sporothrix* species also display phenotypical differences, such as host range, virulence, sensitivity to antifungal drugs, and their interaction with host immunity [14,15,16,17]. *S. schenckii*, *S. brasiliensis*, and *S. globosa* have been isolated from humans, animals, decomposing organic matter, soil, leaves, and wood, indicating they follow an infection route through implantation during the saprophytic phase via contaminated material and the development of the parasitic phase inside the host, with morphological switching being an important virulence factor [14,18,19,20]. However, *S. brasiliensis* has also been associated with zoonotic transmission through bites and scratches from infected animals and has a high prevalence in cats [21,22,23], and thus, it is considered a hyperendemic condition in Brazil [24]. Virulence analysis has shown that *S. brasiliensis* is the most virulent species, followed by *S. schenckii* and *S. globosa* [15,16,25].

The host immune response during sporotrichosis has been more studied in *S. schenckii*- and *S. brasiliensis*-associated diseases, whilst information about *S. globosa* immune sensing is currently scarce and many details remain unknown [10,26,27]. Both functional humoral immunity and cellular immunity are paramount to controlling fungal pathogens [27,28]; like in other fungal species, the *Sporothrix* innate immune response is mediated by pathogen-associated molecular patterns (PAMPs), which are recognized by pattern recognition receptors (PRRs) and are expressed in virtually all the innate immune cell types [17,27,28,29].

The cell wall is the main source of PAMPs and in *Sporothrix*, this structure contains chitin, β-glucans, melanin, glycoproteins, and peptidorhamnomannans, which are directly involved in fungal innate immune sensing [15,17,30,31,32,33]. Although *S. schenckii* and *S. brasiliensis* cell walls contain the same components, these are found in different proportions and distributions within the wall, contributing to the species-specific immune sensing [15,17,30,31,32]; in addition, changes observed in the wall composition depend on the fungal cell morphology [17]. TLR2 and TLR4 are PRRs that have been reported to recognize the different morphotypes of *S. schenckii* and *S. brasiliensis*, and both receptors are required for cytokine stimulation during the interaction of human peripheral blood mononuclear cells (PBMCs) with both fungal species [17]. Dectin-1, a C-type lectin receptor that binds to β-1,3-glucans, is essential for cytokine production during the interaction of human PBMCs with *S. schenckii* and *S. brasiliensis* [17], but the mannose receptor (MR) is relevant for cytokine production only when the human cells are challenged with either *S. schenckii* conidia or *S. brasiliensis* yeast-like cells [17]. However, recent work has shown that the interaction of keratinocytes with *S. schenckii* conidia overexpress MR, TLR6, and TLR2 [34]. MR overexpression could be indicative of the active participation of this receptor in pathogen sensing, as described for *Candida albicans* [34].

Thus far, the study of the innate immune response against *S. globosa* has been limited. Therefore, we performed a cell wall analysis of this species and established the cytokine profile by using different fungal morphologies to stimulate human PBMCs. We also established the contribution of some PRRs during these interactions and performed back-to-back comparative analyses with human cells stimulated with either *S. schenckii* or *S. brasiliensis*.

## 2. Materials and Methods

### 2.1. Organisms and Growth Conditions

The strains used in this study are *S. schenckii* 1099-18 (ATCC MYA 4821), *S. brasiliensis* 5110 (ATCC MYA 4823), and *S. globosa* FMR 9624. The three strains are clinical isolates [35,36], and the genomes of the *S. schenckii* and *S. brasiliensis* isolates have been previously sequenced [37]. Cells were maintained and propagated at 28 °C in YPD plates (2% (*w/v*) gelatin peptone, 1% (*w/v*) yeast extract, 3% (*w/v*) glucose, and 2% (%) agar). After seven days of incubation, conidia were scrapped off of the agar surface and used to inoculate 20 mL of fresh YPD broth with a pH of 7.8, with a final concentration of 1 × 10^6^ conidia mL^−1^. The cultures were incubated in orbital shakers at 37 °C and 120 rpm for 18 h. Then, aliquots of 10 mL were removed from these cultures and used to grow germling and yeast-like cells in YPD media, as described in [17,38]. To prepare heat-killed (HK) cells, cells were incubated at 60 °C for 2h, as reported in [17]; meanwhile, UV-killed cells were prepared by exposing cells to 4 doses of UV radiation (100 mJ cm^−2^) in a UV-DNA crosslinker CL-3000 (Analytik Jena, Upland, CA, USA). For all cases, the viability loss was confirmed by growing cells on YPD plates at 28 °C for seven days.

### 2.2. Cell Wall Analysis

The cell wall basic sugar composition was determined from the purified wall of cell homogenates generated in a Braun homogenizer, as reported in [17,39]. The soluble fraction was separated from the walls by centrifuging at 8000× *g* for 15 min. The pellet was saved, washed six times with deionized water, and subjected to a series of incubations with hot SDS, β-mercaptoethanol, and NaCl to remove intracellular components, as described in [40]. Walls were hydrolyzed with 2 M of trifluoroacetic acid (Sigma-Aldrich, St Louis, MO, USA) and analyzed by high-performance anion-exchange chromatography with pulsed amperometric detection (HPAEC-PAD) in a Dionex system (Thermo Fisher Scientific, Waltham, MA, USA) equipped with a CarboPac PA-1 column and using the elution conditions described elsewhere [41].

Cell wall porosity was determined by assessing the accessibility of DEAE-dextran to the plasma membrane and using the porosity of poly-L-lysine to normalize the data [42]. Aliquots of 1 × 10^8^ cells were suspended in 10 mM of Tris-HCl with a pH of 7.4 (buffer A), buffer A plus 30 mg mL^−1^ of DEAE-dextran (MW. 500 kDa, Sigma-Aldrich), or buffer A plus 30 mg mL^−1^ of poly-L-lysine (MW. 30–70 kDa, Sigma-Aldrich), and incubated for 30 min at 30 °C with shaking at 200 rpm. Preparations were centrifuged at 11,000× *g* for 2 min at room temperature and the supernatant was saved to be further centrifuged twice under the same conditions, using it to measure the absorbance at 260 nm. The 100% porosity corresponds to the 260 nm of leaked material from the treatment with poly-L-lysine [42].

Both *N*-linked and *O*-linked glycan contents of the cell wall were calculated as reported in [43]. The *O*-linked glycans were removed by suspending fungal cells in 0.1 N of NaOH and incubating for 18 h at room temperature and 80 rpm. The *N*-linked glycans were enzymatically trimmed with 25 U of endoglycosidase H (New England Biolabs; Ipswich, MA, USA) and incubated at 37 °C for 20 h. In both cases, the pH was neutralized at the end of the incubation time, and fungal cells were pelleted by centrifuging and used to interact with PBMCs. Glycans were recovered from supernatants and quantification was assessed by HPAEC-PAD, using the separation conditions previously reported [44].

The β-1,3-glucan and chitin exposure at the cell wall surface was analyzed by staining with fluorescence-conjugated lectins and comparing the labeling of live and HK fungal cells. For the β-1,3-glucan labeling, cells were incubated with 5 μg mL^−1^ of IgG Fc-dectin-1 chimera [45] for 40 min at room temperature and then with 1 μg mL^−1^ of donkey anti-Fc IgG-fluorescein isothiocyanate (Sigma-Aldrich) for 40 min at room temperature [46]. For chitin labeling, cells were incubated with 1 mg mL^−1^ of wheat germ agglutinin-fluorescein isothiocyanate (Sigma-Aldrich) for 60 min at room temperature [47]. β-1,3-glucan and chitin were labeled separately. Samples were inspected under fluorescence microscopy using a Zeiss Axioscope-40 microscope and an Axiocam MRc camera. The pictures of three hundred cells per morphology and species were acquired and the median fluorescence was estimated as previously described [48]. The 100% fluorescence corresponds to that calculated in HK cells.

### 2.3. Ethics Statement

The inclusion and use of human cells were approved by the Ethics Committee from the Universidad de Guanajuato (reference CIBIUG-P12-2018). Only healthy adult volunteers were enrolled in the study, and venous blood samples were collected after detailed information about the study was provided and written informed consent was obtained from donors. This procedure was conducted following the Declaration of Helsinki.

### 2.4. Human PBMC–Fungus Interaction

Human PBMCs were isolated from EDTA-treated peripheral blood samples by mixing them with Histopaque-1077 (Sigma-Aldrich) and performing density centrifugation, as previously reported [49]. The mononuclear cells were washed twice in sterile PBS and suspended in RPMI 1640 Dutch modification (Sigma-Aldrich). The immune cell–*Sporothrix* interactions occurred in a volume of 200 μL in 96-well microplates, containing 5 × 10^5^ human cells and 1 × 10^5^ fungal cells. To block specific immune receptors, human PBMCs were pre-incubated for 60 min at 37 °C with 5% (*v/v*) CO_2_ with one of the following compounds before interaction with fungal cells: 200 μg mL^−1^ of laminarin (Sigma-Aldrich) [50], 10 μg mL^−1^ of anti-mannose receptor (MR) (Thermo-Fisher Scientific, MA5-44033), 10 μg mL^−1^ of anti-TLR4 (Santa Cruz Biotechnology, Dallas, TX, sc-293072), or 10 μg mL^−1^ of anti-TLR2 (Thermo-Fisher Scientific, 16-9922-82) [17,51]. As controls, isotype-matched, irrelevant IgG_1_ antibodies (10 μg mL^−1^, Santa Cruz Biotechnology, Cat. No. sc-52003) were used in experiments where MR and TLR4 were blocked; meanwhile, 10 μg mL^−1^ of IgG_2_aκ (Thermo-Fisher Scientific, 14-4724-85) was used as a control in TLR2 blocking experiments. All reagents used were LPS-free, as demonstrated by the *Limulus* amebocyte lysate (Sigma-Aldrich); nevertheless, 5 μg mL^−1^ of polymyxin B (Sigma-Aldrich) was included in all the pre-incubation steps [52]. For tumor necrosis factor-alpha (TNFα), interleukin 1β (IL-1β), interleukin 6 (IL-6), and interleukin 10 (IL-10) stimulation, plates were incubated for 24 h at 37 °C with 5% (*v/v*) CO_2_ [17,48,53]; meanwhile, for the interleukin 17 (IL-17) and interleukin 22 (IL-22) stimulation, the human PBMCs were added to 10% (*v*/*v*) human pooled serum, the interactions were performed with UV-killed fungal cells, and the plates were incubated for 7 days at 37 °C with 5% (*v/v*) CO_2_ [54]. Dead fungi were used to keep the cell morphology and wall organization intact [55]. In addition, the use of UV-killed cells instead of living cells helps to avoid the overgrowth of viable fungi that could affect cytokine production during long co-incubation times [54]. In all cases, plates were centrifuged for 10 min at 1800× *g* at 4 °C, and supernatants were saved and kept at −20 °C until used. The supernatants were used for cytokine measurements. TNFα, IL-6, and IL-10 were quantified via ELISA with Standard ABTS ELISA Development kits from Peprotech; IL-1β, IL-17, and IL-22 were also quantified via ELISA with a DuoSet ELISA Development kit from R&D Systems. For TNFα, IL-1β, IL-6, and IL-10 quantification, wells containing only human PBMCs and the RPMI 1640 Dutch modification (Sigma-Aldrich) were included in all the interactions as controls, whereas for IL-17 and IL-22 stimulation, the control cells were human PBMCs and the RPMI 1640 Dutch modification (Sigma-Aldrich) added to the 10% (*v/v*) human pooled serum. In all cases, the control wells gave threshold values that were subtracted from the different interactions performed on the same plate.

### 2.5. Statistical Analysis

Statistical analysis was performed using GraphPad Prism 6 software and the Mann–Whitney *U* test. Data are cumulative results of all experiments performed and are shown as mean ± S.D. Cytokine stimulation using human innate cells was performed in duplicate with eight healthy donors; meanwhile, other experiments were performed at least three times in triplicate. In all cases, the statistical significance was set at *p* < 0.05.

## 3. Results

### 3.1. The Sporothrix schenckii, Sporothrix brasiliensis, and Sporothrix globosa Cell Wall Is Affected by Cell Morphology

It has been previously reported that the cell walls of *S. schenckii* and *S. brasiliensis* conidia, yeast-like cells, and germlings contain the same structural components but differ in abundance and organization [17,30]. Thus far, the composition of the *S. globosa* yeast-like cells has been reported, but there is no information about it in conidia and germlings [15]. Since in *Sporothrix* spp., such as in other fungal species, the cell wall is the fungal structure that interacts with the host immunity in the first place, we analyzed the cell wall compositions of *S. globosa* conidia, yeast-like cells, and germlings and compared them with the other two clinically relevant species, i.e., *S. schenckii* and *S. brasiliensis*. The cell walls from the three species and the three morphologies showed the same basic components, namely, glucosamine (the building unit of chitin), glucose (the monomer of glucans), rhamnose, and mannose (both composed of rhamnomannan) [15,17,30,31,56,57,58], but their abundance was morphology- and species-dependent (Table 1). In all cases, glucosamine was the monosaccharide that was less abundant, but its concentration was significantly higher in *S. schenckii* germlings when compared with conidia or yeast-like cells from the same species (Table 1). For the case of *S. brasiliensis*, glucosamine was significantly higher in yeast-like cells and germlings, whereas no changes associated with morphology were observed for the glucosamine content in *S. globosa* (Table 1). When compared across species, *S. brasiliensis* conidia and yeast-like cells showed the lowest glucosamine levels, and the *S. globosa* cells had the highest monosaccharide levels (Table 1). The *S. schenckii* germling glucose content was the highest among the three morphologies analyzed, but in *S. brasiliensis* the monosaccharide levels were lower in both yeast-like cells and germlings (Table 1). The glucose content in the *S. globosa* cell wall was not influenced by the cell morphology (Table 1), but the levels associated with conidia and yeast-like cells were the highest when compared to the other two species (Table 1). Mannose content in the *S. schenckii* germling cell wall was significantly low, but no changes in the content of this monosaccharide were observed in the morphologies of *S. brasiliensis* or *S. globosa* (Table 1). However, the mannose content in the *S. globosa* cell walls was the lowest among the three species (Table 1). The *S. schenckii* germling cell wall showed significantly low rhamnose levels; meanwhile, in *S. brasiliensis*, the highest abundance for this monosaccharide was observed in yeast-like cells (Table 1). For the case of *S. globosa*, no significant changes were observed in the three cell morphologies analyzed, but rhamnose content in the three morphologies was significantly different when compared to *S. schenckii* or *S. brasiliensis* (Table 1).

**Figure 1:**
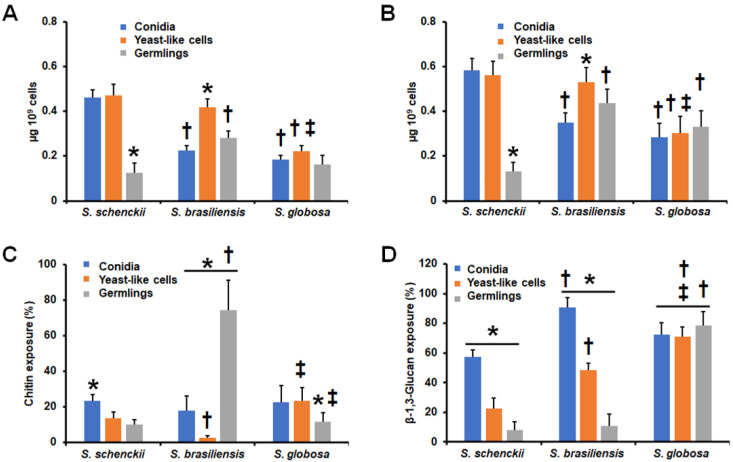
Cell wall analysis of the different *Sporothrix schenckii*, *Sporothrix brasiliensis*, and *Sporothrix globosa* morphologies. In (**A**), cells were treated with 25 U of endoglycosidase H for 20 h at 37 °C and the released *N*-linked glycans were quantified by high-performance anion-exchange chromatography with pulsed amperometric detection (HPAEC-PAD). In (**B**), cells were treated overnight with 0.1 N of NaOH, and the trimmed *O*-linked glycans were saved and quantified by HPAEC-PAD. Cells were stained with 1 mg mL^−1^ of wheat germ agglutinin-fluorescein isothiocyanate (panel (**C**)) or with 5 μg mL^−1^ of IgG Fc-Dectin-1 chimera, and then with 1 μg mL^−1^ of donkey anti-Fc IgG-fluorescein (panel (**D**)) to stain chitin and β-1,3-glucan, respectively. The fluorescence of 300 cells for each condition was calculated and the median fluorescence was estimated as previously. The 100 % fluorescence corresponds to that calculated in heat-killed cells. *S. schenckii*—*Sporothrix schenckii*; *S. brasiliensis*—*Sporothrix brasiliensis*; *S. globosa*—*Sporothrix globosa*. Results are the median ± standard deviation from three independent experiments performed in triplicate. * *p* < 0.05 when compared to other morphologies from the same species; † *p* < 0.05 when compared to the same morphology in the other two fungal species; ‡ *p* <0.05 when compared to the same morphology in *S. brasiliensis*.

Cell wall porosity has been previously associated with the thickness of the layer composed of the oligosaccharides attached to cell wall glycoproteins [17,42,59,60], and thus, we assessed the relative wall porosity compared to that of bulky polycation DEAE-dextran [42]. The *S. schenckii* germlings showed the lowest wall porosity among the three morphologies analyzed (Table 1), but in the case of *S. brasiliensis,* conidia showed the lowest porosity levels (Table 1). No differences were observed in the porosity of the three *S. globosa* morphologies, but these were significantly higher when compared with either *S. schenckii* or *S. brasiliensis* (Table 1). Next, the *N*-linked and *O*-linked glycans from the fungal cell walls were removed by treatment with Endo-H or β-elimination, respectively, and quantified by HPAEC-PAD [44]. The *N*-linked and *O*-linked glycan content of *S. schenckii* germlings was significantly low when compared to the other cell morphologies (Figure 1A,B), and when a similar analysis was performed with the *S. brasiliensis* cell walls, we found that yeast-like cells showed the highest content of both *N*-linked and *O*-linked glycans (Figure 1A,B). In the case of *S. globosa*, the three cell morphologies showed similar levels of both *N*-linked and *O*-linked glycans (Figure 1A,B). However, the *S. globosa N*-linked and *O*-linked glycan contents were lower when compared to that observed in yeast-like cells of the other two fungal species or when compared to *S. schenckii* conidia (Figure 1A,B). For the case of *O*-linked glycans, these were significantly higher in both *S. brasiliensis* and *S. globosa* germlings (Figure 1B).

Structural cell wall polysaccharides, such as chitin and β-1,3-glucan, play a relevant role during the host–fungus interaction, particularly when exposed to the cell surface [15,43,47,48,50,53,55,56,57,60,61]. Therefore, we assessed the exposure of these two cell wall polysaccharides in the cell surfaces of conidia, yeast-like cells, and germlings from the three fungal species. *S. schenckii* conidia showed the highest exposure of both polysaccharides among the three cell morphologies under study (Figure 1C,D), and for the case of β-1,3-glucan exposure, the germlings showed the lowest (Figure 1D). For *S. brasiliensis*, yeast-like cells displayed the lowest amount of chitin on the cell surface, and germlings showed the highest exposure of this polysaccharide at the cell wall surface (Figure 1C). Contrary to the observation with chitin, β-13-glucan exposure showed the highest levels in conidia, followed by in yeast-like cells and germlings (Figure 1D). Finally, for the case of *S. globosa*, no changes in the β-1,3-glucan exposure on the cell surfaces of the three morphologies were observed, and germlings showed a significant reduction in the chitin exposure when compared to conidia or yeast-like cells (Figure 1C,D). However, chitin exposure was significantly different in conidia and yeast-like cells of *S. brasiliensis* and *S. globosa* (Figure 1C), and β-1,3-glucan exposure in the three *S. globosa* morphologies was significantly different when compared to the counterparts in *S. schenckii* and *S. brasiliensis*. Collectively, these data suggest that the cell wall has a species- and morphology-specific composition and organization in *S. schenckii*, *S. brasiliensis*, and *S. globosa.*

### 3.2. Sporothrix schenckii, Sporothrix brasiliensis, and Sporothrix globosa Differentially Stimulate Cytokine Production by Human Peripheral Blood Mononuclear Cells

**Figure 2:**
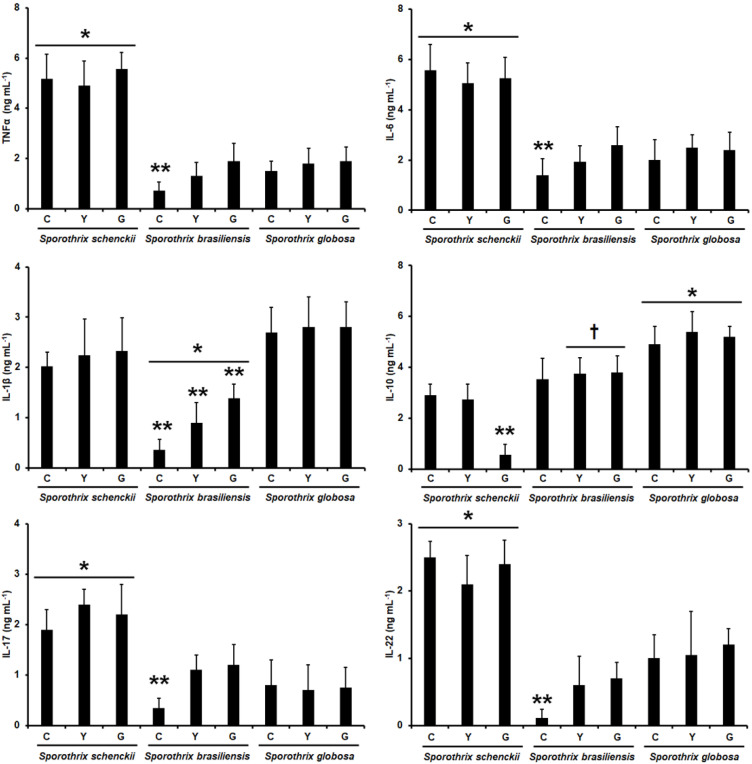
Cytokine production by human peripheral blood mononuclear cells stimulated with *Sporothrix schenckii*, *Sporothrix brasiliensis,* or *Sporothrix globosa*. For tumor necrosis factor-alpha (TNFα), interleukin 1β (IL-1β), interleukin 6 (IL-6), and interleukin 10 (IL-10), human peripheral blood mononuclear cells were co-incubated for 24 h with live conidia, yeast-like cells, or germlings from the fungal species under study. Alternatively, for interleukin 17 (IL-17) and interleukin 22 (IL-22) stimulation, the human cells were co-incubated with the UV-inactivated fungal cells. In all cases, the interactions were centrifuged, supernatants collected, and cytokines quantified by ELISA. C—conidia; Y—yeast-like cells; G—germlings. Results are the median ± standard deviation from data generated with samples from eight donors, analyzed in duplicate. * *p* < 0.05 when compared with the cytokine level stimulated by other *Sporothrix* species; ** *p* < 0.05 when compared with the cytokine levels stimulated by the other morphologies of the same species; † *p* < 0.05 when compared with the cytokine levels stimulated with *S. schenckii* cells.

The human PBMCs’ interaction with conidia, yeast-like cells, and germlings from *S. schenckii* and *S. brasiliensis* has been previously reported, along with the profile of TNFα, IL-1β, IL-6, and IL-10 stimulation. Here, to have a back-to-back comparison, we replicated these previously reported data under the same experimental setting and compared them with those generated with *S. globosa* in its different morphologies. In addition, we expanded the repertoire of cytokines analyzed and included the analysis of the Th17 subset for immunity against *Sporothrix* spp., measuring IL-17 and IL-22 production. Since the stimulation of both cytokines requires incubation periods in the range of days (7 days in our experimental setting) [54], it was not possible to use live cells for the interactions, as the incubation conditions did not allow us to keep cells in a specific fungal morphology (cells tend to grow, generating hyphae and yeast-like cells). Thus, to preserve the fungal morphology and cell wall organization, we used UV-killed cells, which have been reported to lose viability but keep the wall integrity intact [55,62,63]. There was a similar production profile for both TNFα and IL-6 stimulation when using *S. schenckii* conidia, yeast-like cells, or germlings in interactions with the human cells, and the levels obtained with any of these three fungal morphologies were the highest across the three fungal species analyzed (Figure 2). For TNFα and IL-6 production stimulated with *S. brasiliensis* and *S. globosa,* the levels were similar when compared, with the only exception being the cytokine levels stimulated with *S. brasiliensis* conidia, which were significantly lower when compared with the other two morphologies of the same fungal species (Figure 2). The different *S. schenckii* and *S. globosa* morphologies stimulated similar IL-1β levels, and even though the levels associated with the *S. globosa* cells tended to be higher, these were not statistically significant (*p* > 0.5 in all cases; Figure 2). The cells stimulated with *S. brasiliensis* produced the lowest IL-1β levels with a morphology-dependent stimulation, with the lowest, medium, and highest cytokine levels simulated by conidia, yeast-like cells, and germlings, respectively (Figure 2). For the case of IL-10 stimulation, the cytokine was highly produced in cells stimulated with any of the three *S. globosa* morphologies; meanwhile, *S. brasiliensis* cells stimulated lower IL-10 levels when compared to cells incubated with *S. globosa*, but these were significantly higher when compared to those stimulated with *S. schenckii* yeast-like cells or germlings (Figure 2). For the case of *S. schenckii*, conidia and yeast-like cells stimulated similar IL-10 levels, but these were significantly higher when compared with those stimulated with germlings (Figure 2). It is worth mentioning that these cytokine profiles associated with the *S. schenckii* and *S. brasiliensis* morphologies are like those previously reported [17]. Finally, the UV-killed *S. schenckii* cells induced high IL-17 and IL-22 levels, regardless of the cell morphology; meanwhile, cells stimulated with *S. brasiliensis* or *S. globosa* produced lower and similar levels of both cytokines, with the only exception being *S. brasiliensis* conidia that stimulated the lowest levels of both cytokines (Figure 2). Therefore, these data indicate that the three fungal species are differentially recognized by human PBMCs and that the fungal morphology plays a role in such an interaction.

**Figure 3:**
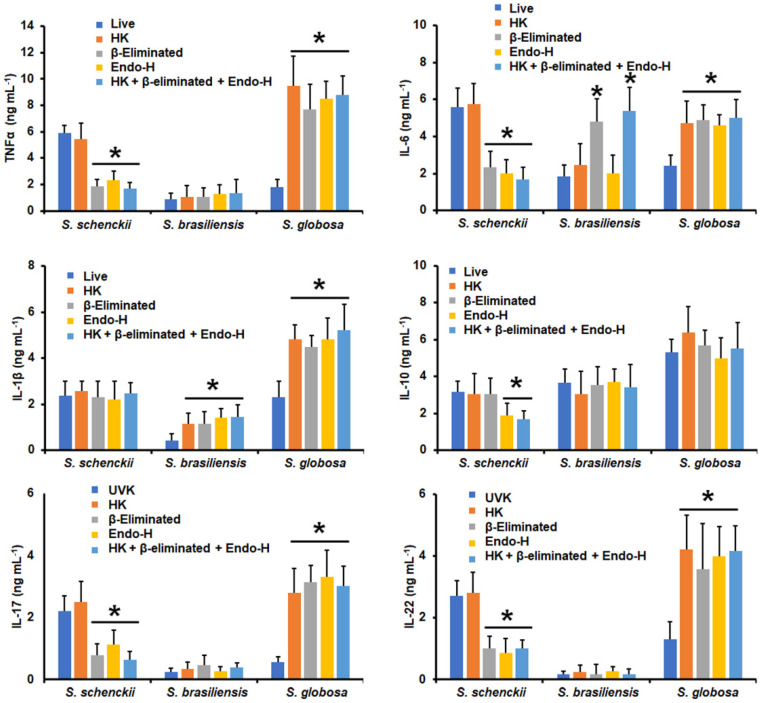
Cytokine production by human peripheral blood mononuclear cells stimulated with conidia from *Sporothrix schenckii*, *Sporothrix brasiliensis*, or *Sporothrix globosa*. For tumor necrosis factor-alpha (TNFα), interleukin 1β (IL-1β), interleukin 6 (IL-6), and interleukin 10 (IL-10), human peripheral blood mononuclear cells were co-incubated for 24 h with conidia previously subjected to the indicated treatments (see Materials and Methods for details). Alternatively, for interleukin 17 (IL-17) and interleukin 22 (IL-22) stimulation, the human cells were co-incubated with the UV-killed fungal cells or subjected to the indicated treatments. In all cases, the interactions were centrifuged, supernatants collected, and cytokines quantified by ELISA. UVK—UV-killed cells; HK—heat-killed cells; Endo-H—treatment with endoglycosidase H. Results are the median ± standard deviation from data generated with samples from eight donors, analyzed in duplicate. * *p* < 0.05 when compared with the cytokine level stimulated by live or UV-killed cells.

Next, we assessed the contribution of some cell wall components of the three fungal species to cytokine stimulation. Cells were heat-killed before the interaction with human PMBCs, as this treatment has been previously demonstrated to artifactually expose inner cell wall components, particularly β-1,3-glucan [17,48,55]. In addition, cells were depleted of either *N*-linked glycans or *O*-linked glycans by incubation with endoglycosidase H or β-elimination, respectively [43,51,53], or were subjected to the three consecutive treatments by removing both kinds of glycans and exposing the inner wall components. The cytokine production stimulated by *S. schenckii* conidia was not affected by heat killing, suggesting inner wall components are dispensable for cytokine stimulation (Figure 3). However, a significant reduction in the TNFα, IL-6, IL-17, and IL-22 levels was observed upon removal of either the *N*-linked glycans or *O*-linked glycans (Figure 3). IL-10 stimulation was only sensitive to the removal of *N*-linked glycans, whereas IL-1β production was not affected by these treatments (Figure 3). For *S. brasiliensis* conidia, none of the treatments affected the ability to stimulate TNFα, IL-10, IL-17, and IL-22, but β-elimination significantly increased IL-6 production, and heat killing, β-elimination, and the Endo-H treatment positively affected IL-1β stimulation (Figure 3). *S. globosa* conidia stimulated significantly higher levels of all the proinflammatory cytokines tested when cells were heat-killed, β-eliminated, or treated with Endo-H (Figure 3); meanwhile, IL-10 production did not change when human cells were challenged with fungal cells treated with any of the above-mentioned treatments (Figure 3).

**Figure 4:**
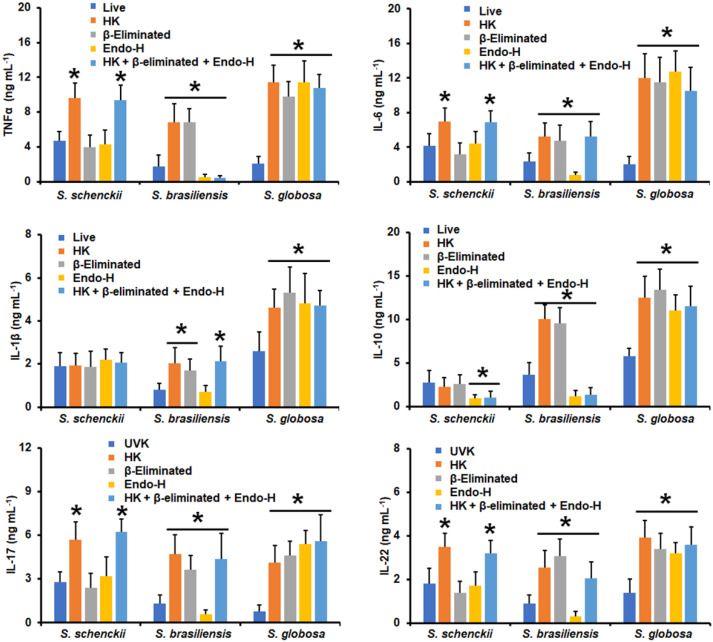
Cytokine production by human peripheral blood mononuclear cells stimulated with yeast-like cells from *Sporothrix schenckii*, *Sporothrix brasiliensis*, or *Sporothrix globosa*. For tumor necrosis factor-alpha (TNFα), interleukin 1β (IL-1β), interleukin 6 (IL-6), and interleukin 10 (IL-10), human peripheral blood mononuclear cells were co-incubated for 24 h with conidia previously subjected to the indicated treatments (see Materials and Methods for details). Alternatively, for interleukin 17 (IL-17) and interleukin 22 (IL-22) stimulation, the human cells were co-incubated with the UV-killed fungal cells or subjected to the indicated treatments. In all cases, the interactions were centrifuged, supernatants collected, and cytokines quantified by ELISA. UVK—UV-killed cells; HK—heat-killed cells; Endo-H—treatment with endoglycosidase H. Results are the median ± standard deviation from data generated with samples from eight donors, analyzed in duplicate. * *p* < 0.05 when compared with the cytokine level stimulated by live or UV-killed cells.

When yeast-like cells were used in similar experiments, HK *S. schenckii* cells stimulated higher TNFα, IL-6, IL-17, and IL-22 levels, and cells subjected to the three treatments behaved in a similar way (Figure 4). IL-1β stimulation was not sensitive to the cell treatments and IL-10 level, as it was reduced only when the *N*-linked glycans were removed (Figure 4). *S. brasiliensis* yeast-like cells stimulated higher TNFα levels when HK or β-eliminated, but the cytokine levels were significantly reduced upon treatment with Endo-H or in cells subjected to the three treatments (Figure 4). IL-6, IL-1β, IL-17, and IL-22 levels were higher when human PBMCs were stimulated via HK, β-elimination, or cells subjected to the three treatments, but were diminished when the *N*-linked glycans were removed from yeast-like cells (Figure 4). In the case of IL-10 production, this was only positively affected when cells were HK or β-eliminated (Figure 4). The *S. globosa* yeast-like cells stimulated higher TNFα, IL-6, IL-1β, IL-10, IL-17, and IL-22 levels when cells were subjected to any of the applied treatments when compared to the UV-killed cells (Figure 4).

**Figure 5:**
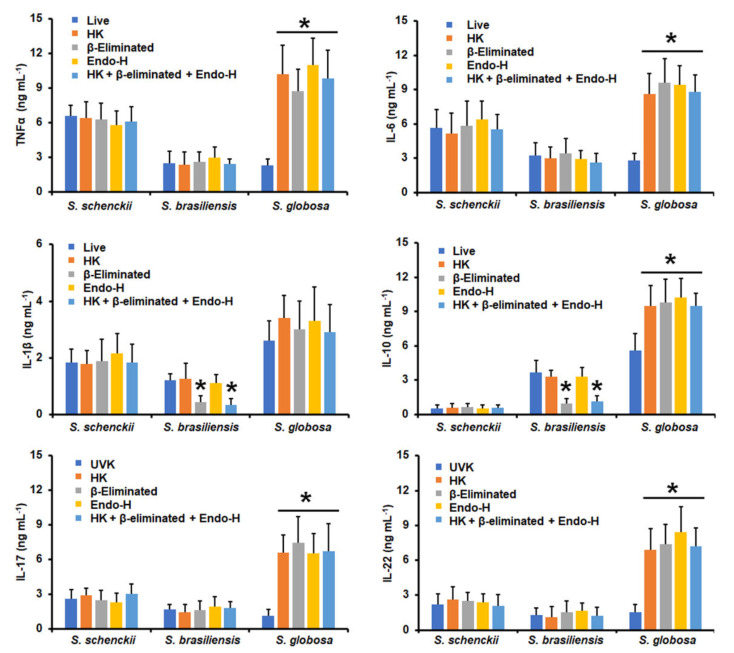
Cytokine production by human peripheral blood mononuclear cells stimulated with germlings from *Sporothrix schenckii*, *Sporothrix brasiliensis*, or *Sporothrix globosa*. For tumor necrosis factor-alpha (TNFα), interleukin 1β (IL-1β), interleukin 6 (IL-6), and interleukin 10 (IL-10), human peripheral blood mononuclear cells were co-incubated for 24 h with conidia previously subjected to the indicated treatments (see Materials and Methods for details). Alternatively, for interleukin 17 (IL-17) and interleukin 22 (IL-22) stimulation, the human cells were co-incubated with the UV-killed fungal cells or subjected to the indicated treatments. In all cases, the interactions were centrifuged, supernatants collected, and cytokines quantified by ELISA. UVK—UV-killed cells; HK—heat-killed cells; Endo-H—treatment with endoglycosidase H. Results are the median ± standard deviation from data generated with samples from eight donors, analyzed in duplicate. * *p* < 0.05 when compared with the cytokine level stimulated by live or UV-killed cells.

*S. schenckii* germlings did not change their ability to stimulate any of the tested cytokines when cells were HK, β-eliminated, or treated with Endo-H (Figure 5). β-Elimination negatively affected the *S. brasiliensis* germlings’ ability to stimulate IL-1β and IL-10, but the other cytokines levels were not affected (Figure 5). Finally, HK, β-elimination, the Endo-H treatment, and the combination of the three treatments positively affected the *S. globosa* germlings to stimulate TNFα, IL-6, IL-10, IL-17, and IL-22 levels, but not IL-1β (Figure 5).

### 3.3. Dectin-1, Mannose Receptor, TLR2, and TLR4 Have Differential Roles during Sporothrix spp.–Human Peripheral Blood Mononuclear Cell Interaction

**Figure 6:**
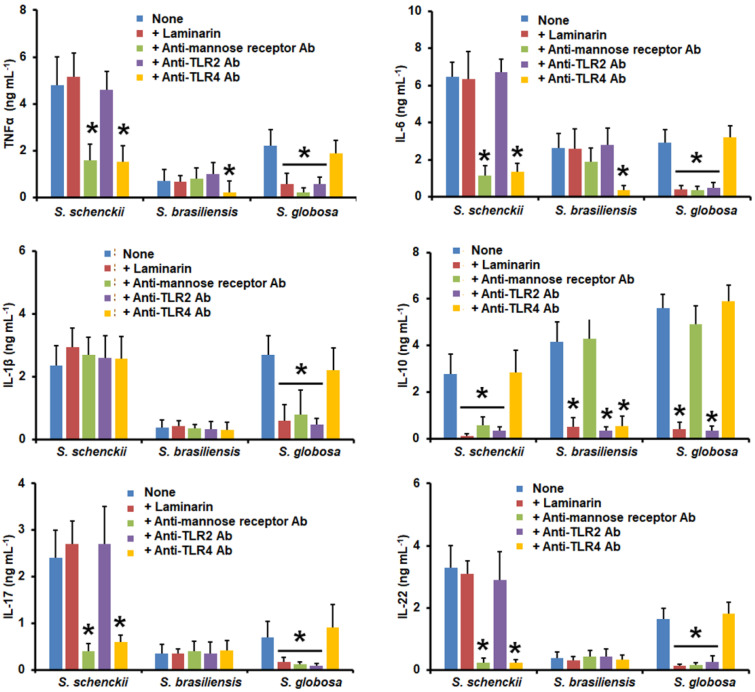
Cytokine production by human peripheral blood mononuclear cells pre-incubated with different blocking molecules and stimulated with conidia from *Sporothrix schenckii*, *Sporothrix brasiliensis*, or *Sporothrix globosa*. Human peripheral blood mononuclear cells were pre-incubated for 60 min with 200 μg mL^−1^ of laminarin, 10 μg mL^−1^ of anti-mannose receptor, 10 μg mL^−1^ of anti-TLR2, or 10 μg mL^−1^ of anti-TLR2, and then challenged with conidia. For tumor necrosis factor-alpha (TNFα), interleukin 1β (IL-1β), interleukin 6 (IL-6), and interleukin 10 (IL-10), human peripheral blood mononuclear cells were co-incubated for 24 h with live conidia, whilst for interleukin 17 (IL-17) and interleukin 22 (IL-22) stimulation, the human cells were co-incubated with the UV-killed conidia. In all cases, the interactions were centrifuged, supernatants collected, and cytokines quantified by ELISA. Ab—antibody; *S.*—*Sporothrix*. None refers to the system where the human cells were pre-incubated only with PBS. Results are the median ± standard deviation from data generated with samples from eight donors, analyzed in duplicate. * *p* < 0.05 when compared with the cytokine level stimulated by PBS-pre-incubated cells.

Next, we assessed the contribution of some pattern recognition receptors to the *Sporothrix*–human PBMC interaction. We particularly focused on dectin-1, MR, TLR2, and TLR4, because these surface receptors have been previously demonstrated to play a role in *S. schenckii* and *S. brasiliensis* immune sensing [17,27,32,64,65,66,67,68]. The TNFα, IL-6, IL-17, and IL-22 stimulation by *S. schenckii* conidia was not affected by laminarin, a specific blocking agent for dectin-1 [69,70], nor by the blocking of TLR2 with specific anti-TLR2 antibodies, but the cytokine levels were significantly reduced when PBMCs were pre-incubated with either anti-MR or anti-TLR4 antibodies (Figure 6). For the IL-10 stimulation, this was affected when dectin-1, MR, or TLR2 were blocked, and no effects of the blocking agents were documented for IL-1β production (Figure 6). When cells were challenged with *S. brasiliensis* conidia, IL-1β, IL-17, and IL-22 levels were not affected by any of the blocking molecules, and only the pre-incubation with anti-TLR4 antibodies significantly affected the TNFα and IL-6 stimulation (Figure 6). IL-10 production was dependent on the blocking of dectin-1, TLR2, or TLR4 (Figure 6). For the case of human cells co-incubated with *S. globosa* conidia, the blocking of dectin-1, MR, or TLR2 significantly reduced the levels of TNFα, IL-6, IL-1β, IL-17, and IL-22; meanwhile, IL-10 production was only reduced when dectin-1 or TLR2 was blocked. No apparent role for TLR4 on the analyzed cytokines was observed when *S. globosa* and immune cells were co-incubated (Figure 6).

**Figure 7:**
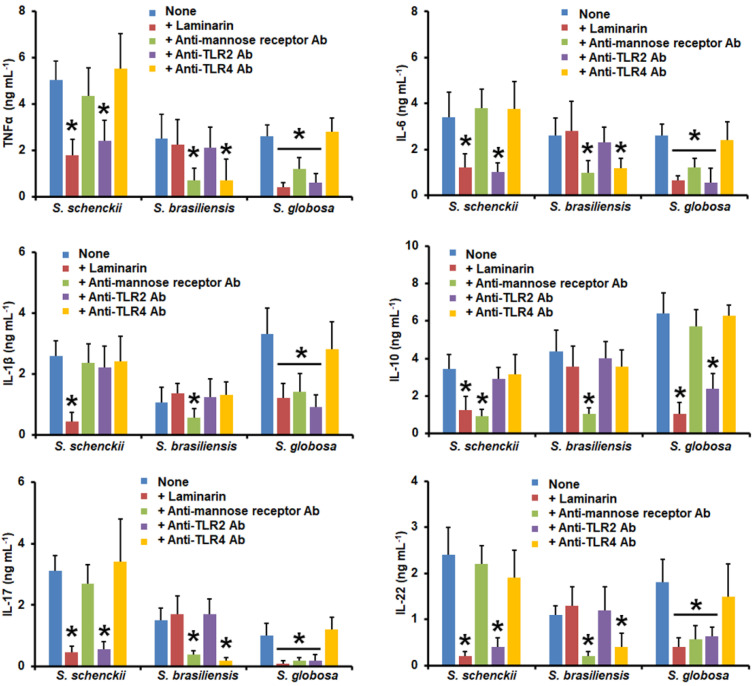
Cytokine production by human peripheral blood mononuclear cells pre-incubated with different blocking molecules and stimulated with yeast-like cells from *Sporothrix schenckii*, *Sporothrix brasiliensis*, or *Sporothrix globosa*. Human peripheral blood mononuclear cells were pre-incubated for 60 min with 200 μg mL^−1^ of laminarin, 10 μg mL^−1^ of anti-mannose receptor, 10 μg mL^−1^ of anti-TLR2, or 10 μg mL^−1^ if anti-TLR2, and then challenged with yeast-like cells. For tumor necrosis factor-alpha (TNFα), interleukin 1β (IL-1β), interleukin 6 (IL-6), and interleukin 10 (IL-10), human peripheral blood mononuclear cells were co-incubated for 24 h with live yeast-like cells, whilst for interleukin 17 (IL-17) and interleukin 22 (IL-22) stimulation, the human cells were co-incubated with the UV-killed fungal cells. In all cases, the interactions were centrifuged, supernatants collected, and cytokines quantified by ELISA. Ab—antibody; *S.*—*Sporothrix*. None refers to the system where the human cells were pre-incubated only with PBS. Results are the median ± standard deviation from data generated with samples from eight donors, analyzed in duplicate. * *p* < 0.05 when compared with the cytokine level stimulated by PBS-pre-incubated cells.

When similar experiments were performed using *S. schenckii* yeast-like cells, TNFα, IL-6, IL-17, and IL-22 levels were only affected by the blocking of dectin-1 or TLR2 (Figure 7); meanwhile, IL-1β was dependent only on the engagement of dectin-1 with its ligand, and for the case of IL-10 production, this was sensitive when dectin-1 or MR was blocked (Figure 7). The MR and TLR4 were required for TNFα, IL-6, IL-17, and IL-22 stimulation by *S. brasiliensis* yeast-like cells, but IL-1β and IL-10 levels were only affected when MR was blocked (Figure 7). When *S. globosa* yeast-like cells were used to challenge the preincubated human PMBCs, we observed that TNFα, IL-6, IL-1β, IL-17, and IL-22 production was dependent on the blocking of dectin-1, MR, or TLR2 (Figure 7). For IL-10 stimulation, this was affected when cells were pre-incubated with either laminarin or anti-TRL2 antibodies (Figure 7).

**Figure 8:**
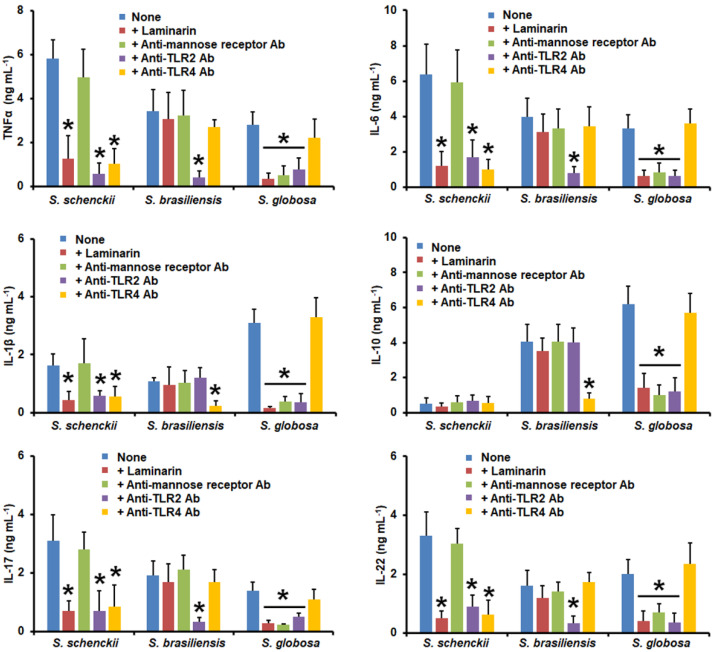
Cytokine production by human peripheral blood mononuclear cells pre-incubated with different blocking molecules and stimulated with germlings from *Sporothrix schenckii*, *Sporothrix brasiliensis*, or *Sporothrix globosa*. Human peripheral blood mononuclear cells were pre-incubated for 60 min with 200 μg mL^−1^ of laminarin, 10 μg mL^−1^ of anti-mannose receptor, 10 μg mL^−1^ of anti-TLR2, or 10 μg mL^−1^ of anti-TLR2, and then challenged with germlings. For tumor necrosis factor-alpha (TNFα), interleukin 1β (IL-1β), interleukin 6 (IL-6), and interleukin 10 (IL-10), human peripheral blood mononuclear cells were co-incubated for 24 h with live germlings, whilst for interleukin 17 (IL-17) and interleukin 22 (IL-22) stimulation, the human cells were co-incubated with the UV-killed fungal cells. In all cases, the interactions were centrifuged, supernatants collected, and cytokines quantified by ELISA. Ab—antibody; *S.*—*Sporothrix*. None refers to the system where the human cells were pre-incubated only with PBS. Results are the median ± standard deviation from data generated with samples from eight donors, analyzed in duplicate. * *p* < 0.05 when compared with the cytokine level stimulated by PBS-pre-incubated cells.

TNFα, IL-6, IL-1β, IL-17, and IL-22 levels stimulated by *S. schenckii* germlings were reduced when dectin-1, TLR2, or TLR4 were blocked; meanwhile, there was no effects observed on the IL-10 production with any of the blocking agents tested (Figure 8). In the case of *S. brasiliensis* germlings, TLR2 blocking negatively affected TNFα, IL-6, IL-17, and IL-22 production, whereas the TLR4 blocking affected IL-1β and IL-10 stimulation (Figure 8). Finally, when *S. globosa* germlings were used to challenge pre-incubated human PBMCs, a significant reduction in all the analyzed cytokines was observed when cells were pre-incubated with laminarin, anti-MR, or anti-TLR2 antibodies (Figure 8). In all cases, control assays with irrelevant isotype-matched antibodies showed similar levels as in the system where PBMCs were pre-incubated with PBS. Collectively, these data indicate that the analyzed receptors have a differential role in cytokine stimulation when interacting with the different morphologies of *S. schenckii*, *S. brasiliensis*, and *S. globosa*.

## 4. Discussion

Thus far, *S. globosa* is the less studied member of the *Sporothrix* pathogenic clade [10], and most of the information related to its interaction with host immunity is extrapolated from data generated with *S. schenckii* or *S. brasiliensis*. Therefore, this work aimed to compare the cell wall composition and the relevance of some of its components during its interaction with human PBMCs. The inclusion of *S. schenckii* and *S. brasiliensis* cells in our study provided the advantage that all cells were treated in the same way, and thus, technical bias by the use of different protocols to prepare cells or perform the interactions was minimized. In addition, this helped to validate our previous study on the *S. schenckii* and *S. brasiliensis* cell wall and immune sensing [17].

Here, the analysis of the *S. schenckii* and *S. brasiliensis* cell wall composition under their three cell morphologies was similar to that previously reported [17]. *S. globosa* showed a higher amount of cell wall glucosamine, i.e., chitin, when compared to the other two species, but this was mostly in the inner cell wall; the only exception was in yeast-like cells, where this was more exposed. Higher exposure to chitin in the fungal cell wall may be related to a lower virulence, since it has been reported that chitin-rich heteroglycan extracted from *S. schenckii* protects the host against infection caused by this species by increasing fungal clearance, phagocytosis, and the production of pro- and anti-inflammatory cytokines to modulate the immune response [71]. Even though we did not address melanin production in the cell preparation used in this study, it is worth noting *S. globosa* melanin inhibits the expression of antigen presentation-associated molecules and fungal phagocytosis [72] and protects against oxygen and nitrogen radicals, decreasing TNFα and IL-6 production [73], which is in concordance with the cytokine profile reported here for the three *S. globosa* morphologies.

Similarly, glucan content was higher in this organism, and most of the β-1,3-glucans were exposed at the cell wall surface, contrary to what was observed in *S. schenckii* and *S. brasiliensis* germlings. With these results, it is possible to hypothesize that the contribution of β-1,3-glucans to *S. globosa* immune sensing could be different than in *S. schenckii* and *S. brasiliensis*. Regarding the outermost part of the cell wall, which is composed of mannose- and rhamnose-based glycoconjugates [17,30], the fact that both sugars were present in low levels in the *S. globosa* cell wall and that the *N*-linked glycans contributed most of the *S. schenckii* cell wall mannose and rhamnose [56,57] suggests that *N*-linked glycans are less abundant in *S. globosa* than in *S. schenckii* or *S. brasiliensis*. This hypothesis is strengthened by our observation here that the three *S. globosa* morphologies had lower *N*-linked glycan levels than their counterparts in the other two species under analysis. A similar rationale can be used to explain the low levels of *O*-linked glycans found in the *S. globosa* cell wall. Cell wall porosity has been previously used to analyze the thickness of the outermost wall layer [43,48,50,53,59,60,61]; according to the low levels of *N*-linked and *O*-linked glycans on the *S. globosa* cell wall, this is highly porous to DEAE-dextran, suggesting that this part of the cell wall is not as dense as in *S. schenckii* or *S. brasiliensis*. Altogether, these results indicate that the *S. globosa* cell wall has species-specific proportions and organization of wall components.

**Figure 9:**
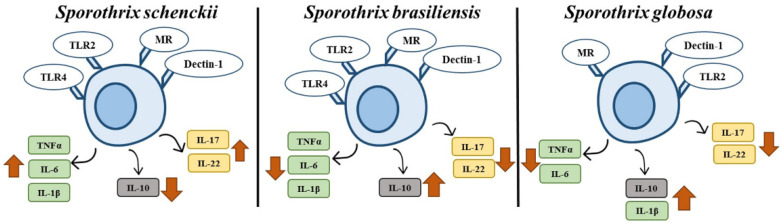
Cytokine profiles of human peripheral blood mononuclear cells stimulated with *Sporothrix schenckii*, *Sporothrix brasiliensis*, or *Sporothrix globosa*. When human cells interact with *S. schenckii*, there is an increased production of proinflammatory cytokines and low IL-10 stimulation. In the case of *S. brasiliensis*, there is a low stimulation of proinflammatory cytokines but high levels of IL-10. A similar cytokine profile was observed when human PBMCs were stimulated with *S. globosa,* with the exception of IL-1β. The receptors involved in the sensing of these three fungal species are mannose receptor (MR), TLR2, dectin-1, and TLR4; however, this last one is dispensable for cytokine stimulation by *S. globosa*.

The cytokine profile associated with the different *S. schenckii* and *S. brasiliensis* morphotypes showed similarities with that previously generated by our groups [17], where *S. schenckii* is capable of inducing a more robust proinflammatory response than *S. brasiliensis* and *S. globosa* (Figure 9). Our results with *S. schenckii* contrast with those recently published [32], and this is likely explained by the use of different culture media and protocols to heat-kill cells and perform the cell–cell interactions. These are aspects that, when modified, affect the *Sporothrix* cell wall and the interaction with the host [15]. These discrepancies underscore the need for a back-to-back experimental setting to obtain fair comparisons between fungal species. With the exception of IL-1β stimulation, the cytokine profiles stimulated by *S. globosa* and *S. brasiliensis* are similar, with high levels of IL-10 (Figure 9). Since low levels of proinflammatory cytokines have been associated with longer survival times in experimental models of candidiasis [74], it is tempting to speculate that this cytokine profile may contribute, along with the poor setting of virulence factors [10], to the low virulence of *S. globosa*.

It has been recently reported that the blockade of complement receptor-3 significantly reduced the ability of *S. schenckii* and *S. brasiliensis* yeast-like cells to stimulate the IL-1β secretion by human monocyte-derived macrophages, and cell wall peptidorhamnomannan could be the fungal ligand for this receptor [75]. Here, no correlation between the mannose + rhamnose content and IL-1 β levels was observed. Similarly, no correlation was obtained between the cell wall glycan content and quantified levels of this cytokine stimulated by yeast-like cells. Thus, these results support the idea that the structure rather than the quantity of cell wall peptidorhamnomannans is behind the reported observations [75]. The combination of β-elimination and the treatment with endoglycosidase H is sufficient to remove most of the cell wall glycans [17,57], including the peptidorhamnomannans [33], and interestingly, cells depleted of cell wall glycans (HK + β-eliminated + endo H in Figure 4) showed different IL-1β stimulation trends: *S. schenckii* yeast-like cells were insensitive to these treatments, but *S. brasiliensis* and *S. globosa* stimulated higher cytokine levels than the untreated cells, suggesting that the role of the complement receptor-3 is different during the sensing of these three fungal species, making it dispensable in the case of the interaction of human PBMCs with either *S. brasiliensis* or *S. globosa* yeast-like cells.

Th17 cells are potent inducers of tissue inflammation, as reported in infection caused by bacteria and fungi [76], and the Th17 response is necessary for host protection during interactions with *S. schenckii* [77]. When the production of IL-17 and IL-22 induced by the different morphotypes of the three *Sporothrix* species was analyzed, it was observed that *S. schenckii* induced higher levels of both cytokines when compared to the other species. These results may indicate that *S. schenckii* is easily recognized by Th17 cells and that this recognition facilitates the elimination of the pathogen, as reported in murine models [78]. The opposite scenario may explain the high virulence observed with *S. brasiliensis*, and again, *S. globosa*’s poor setting of virulence factors may help us to understand why this fungal species is not as successful as *S. brasiliensis* in damaging the host. It is noteworthy to mention that our results are in line with a previous analysis of cytokine production in patients with *S. globosa*-caused sporotrichosis, where reduced serum IL-17 levels and defective Th17 function were reported [79].

Interestingly, when inner wall components were exposed at the *S. globosa* cell surface or glycans were removed, the ability to stimulate cytokine production was similar for these cells, regardless of the morphology. This contrasts with the observations in *S. schenckii* and *S. brasiliensis*, where some effects could be observed depending on the cell treatment or morphology. This increase in the ability of treated cells to stimulate pro-and anti-inflammatory cytokines suggests that: (1) *N*-linked and *O*-linked glycans make a minor contribution to *S. globosa* sensing with human PBMCs; (2) inner wall components, such as, most likely, β-1,3-glucans, are the main stimuli for cytokine stimulation; and (3) the *S. globosa* cell wall plasticity is similar in the three morphologies. This last observation was already reported for yeast-like cells growing on different culture media [15].

The use of laminarin and antibodies against MR, TLR2, and TLR4 indicated that except for TLR4, the other receptors play an important role in *S. globosa* sensing (Figure 9). This minor contribution of TLR4 may be explained by the fact that rhamnose has been reported as one of the *S. schenckii* ligands for this receptor [56], and the low rhamnose levels in *S. globosa* may account for the dispensability of this PRR. The cytokine profile obtained with the different blocking agents in cells challenged with *S. schenckii* or *S. brasiliensis* is similar to that already reported [17], but in *S. globosa* there is no information regarding the receptors involved in the process of its immune recognition. The use of laminarin to block dectin-1 indicated that this receptor is key for cytokine production stimulated by conidia, yeast-like cells, and germlings, and this is in agreement with the observation that the cell wall has high β-1,3-glucan levels exposed at the cell surface. A similar observation for the central role of β-1,3-glucans has been reported for the *S. globosa*–*Galleria mellonella* interaction [15]. In the case of MR, it was observed that this receptor also contributes to the production of proinflammatory cytokines stimulated by *S. globosa* germlings, yeast-like cells, and conidia. The ligand for MR in other fungal species is *N*-linked mannan [80], and it is feasible to speculate that a similar situation occurs when interacting with *S. globosa*. However, the results with Endo-H suggest this cell wall component plays a minority role in cytokine stimulation. One possible explanation for this discrepancy is that mannose-modified lipids found on the cell wall surface may be the actual ligands for MR, as these have been reported to be part of the *Sporothrix* cell wall [26,81]. Regarding the TLR2 receptor, the results showed that this receptor is as important as dectin-1 for cytokine stimulation by *S. globosa*; however, this observation does not apply to *S. schenckii* and *S. brasiliensis*. TLR2 can cooperate with TLR1, TLR6, and dectin-1 to induce cytokine production [82,83]. Thus, it is likely that the relevance of this receptor during the interaction with the different morphologies and *Sporothrix* species depends on having a partner to induce cytokine production. For the case of *S. globosa*, we propose a collaborative stimulation between dectin-1 and TLR2, because it was required that both receptors were accessible in all the experimental conditions.

In conclusion, we found that the cell wall composition and structure of *S. schenckii, S. brasiliensis*, and *S. globosa* are different in conidia, yeast-like cells, and germlings, and the interaction of these cells with human PBMCs generates species-specific cytokine profiles. For the case of *S. globosa,* this tends to stimulate an anti-inflammatory cytokine profile that depends on sensing with dectin-1, MR, and TLR2, but not with TLR4.

## Figures and Tables

**Table 1:** Cell wall composition and porosity of conidia, yeast-like cells, and germlings from *Sporothrix schenckii*, *Sporothrix brasiliensis*, and *Sporothrix globosa*.

Cell Wall Abundance					Porosity (%) ^‖^
Organism	Glucosamine (%)	Mannose (%)	Glucose (%)	Rhamnose (%)	
*Sporothrix schenckii*					
Conidia	6.7 ± 2.9	42.9 ± 6.7	33.3 ± 6.9	17.1 ± 5.1	81.3 ± 6.8
Yeast-like cells	5.7 ± 3.4	41.3 ± 5.2	39.5 ± 4.9	13.5 ± 4.8	78.8 ± 9.7
Germlings	16.9 ± 3.5 *	17.8 ± 1.5 *	60.9 ± 3.7 *	4.4 ± 2.3 *	62.1 ± 4.8 *
*Sporothrix brasiliensis*					
Conidia	2.9 ± 1.4 ^†^	38.8 ± 5.4	50.2 ± 3.7 ^†^	8.1 ± 3.6 ^†^	62.1 ± 10.4 *^†^
Yeast-like cells	5.5 ± 2.7 *	40.7 ± 4.9	22.4 ± 4.7 *^†^	31.4 ± 4.5 *^†^	80.2 ± 9.5
Germlings	5.9 ± 2.7 *^†^	42.5 ± 3.3 ^†^	41.7 ± 4.2 *^†^	9.9 ± 6.4	81.1 ± 10.4 ^†^
*Sporothrix globosa*					
Conidia	9.5 ± 3.2 ^†^	23.6 ± 4.4 ^†^	60.4 ± 3.4 ^†^	6.5 ± 3.4 **	96.5 ± 7.7 ^†^
Yeast-like cells	9.8 ± 4.2 ^†^	30.1 ± 5.8 ^†^	54.7 ± 6.5 ^†^	5.4 ± 4.5 ^†^	98.4 ± 5.1 ^†^
Germlings	10.7 ± 2.8 ^†^	23.8 ± 5.4 ^†^	63.4 ± 6.6 ^‡^	2.1 ± 4.1 ^‡^	95.4 ± 3.8 ^†^

^‖^ Relative to DEAE-dextran, the 100% corresponds to the poly-L-lysine porosity. * *p* < 0.05 when compared to other morphologies of the same species. † *p* < 0.05 when compared to the same morphology of the other two species. ** *p* < 0.05 when compared to the same morphology in *Sporothrix schenckii.* ‡ *p* < 0.05 when compared to the same morphology in *Sporothrix brasiliensis.*

## Data Availability

Not applicable.
